# Supplementary figures and images for: Combining viral genetic and animal mobility network data to unravel peste des petits ruminants transmission dynamics in West Africa
Source: PLoS Pathog. 2021 Mar 18;17(3):e1009397. doi: 10.1371/journal.ppat.1009397 (PMC8009415; doi:10.1371/journal.ppat.1009397)

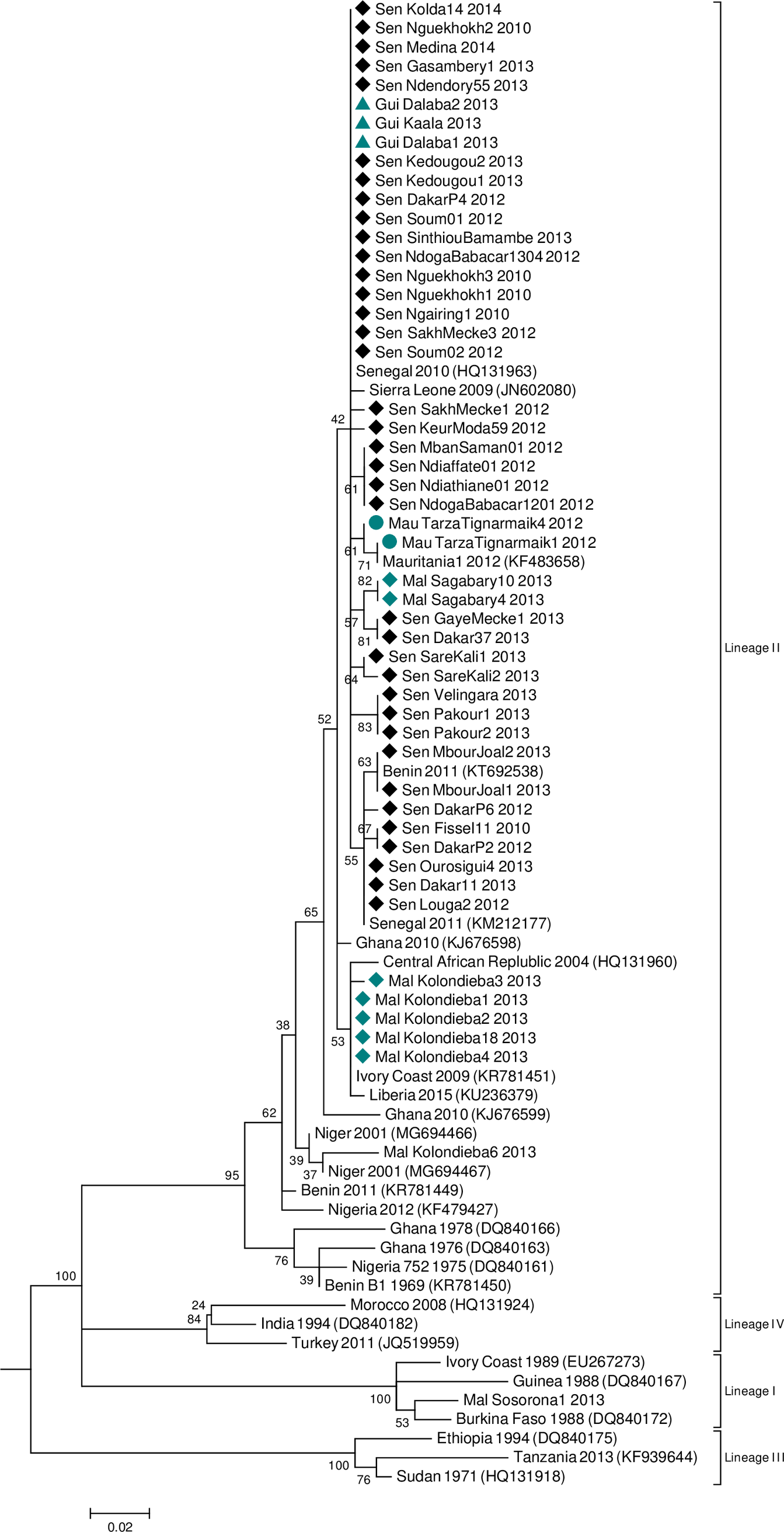

Supplement: S1 Fig — Phylogenetic tree constructed using a maximum likelihood inference method and showing the relationship based on partial N gene sequences (255 bp) of peste des petits ruminants virus (PPRV) samples obtained in this study and publicly available sequences representative of all 4 PPRV genetic lineages. Samples collected in this study are indicated by symbols according to sampling location (Guinea, graphic object Mali, Mauritania, graphic object Senegal). The numbers at the nodes are bootstrap values obtained from 1 000 replicates (Maximum Likelihood methods). Bootstrap values are shown if > 50%. (TIF) [file ppat.1009397.s001.tif]

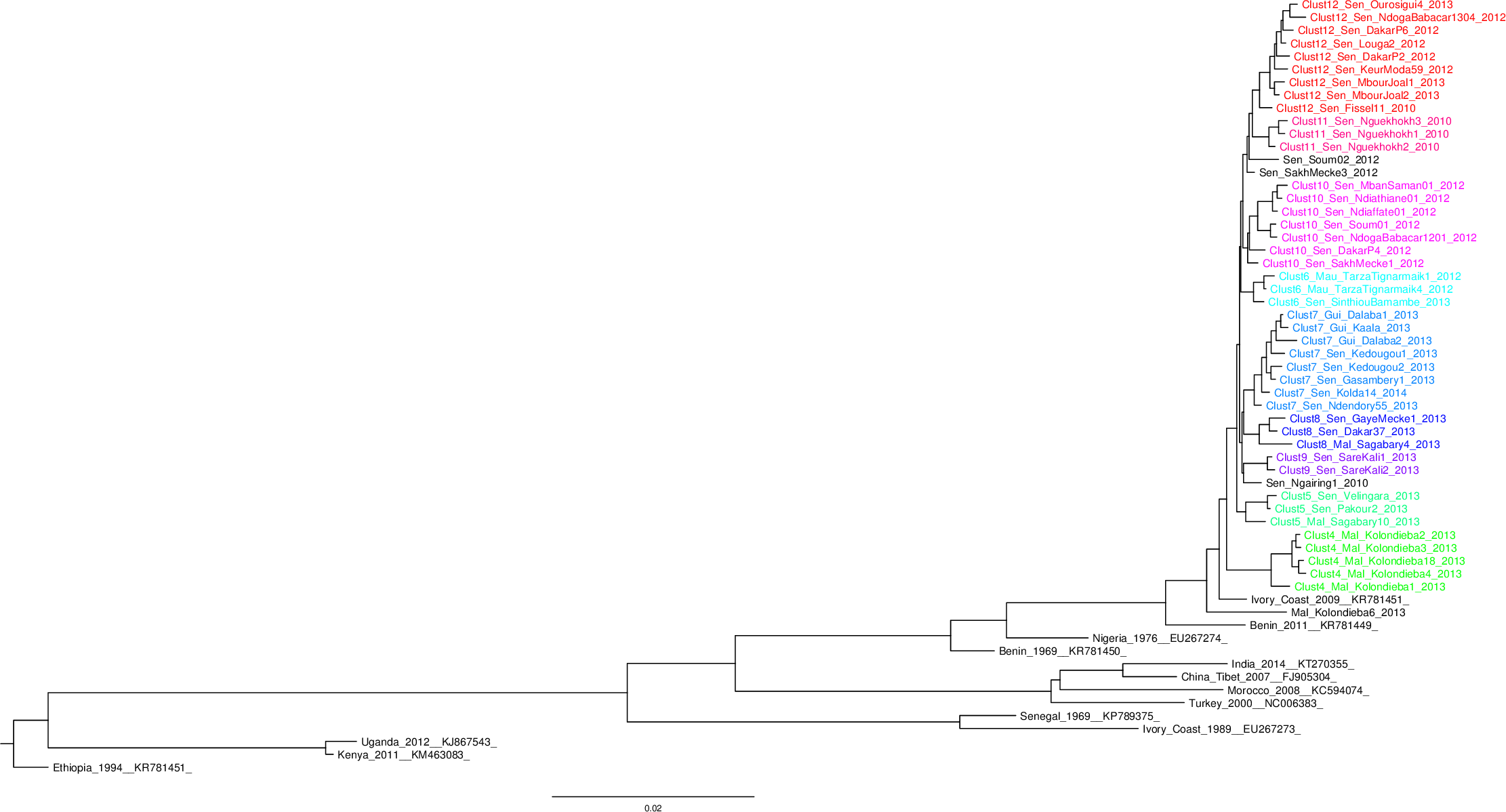

Supplement: S2 Fig — (TIFF) [file ppat.1009397.s002.tiff]

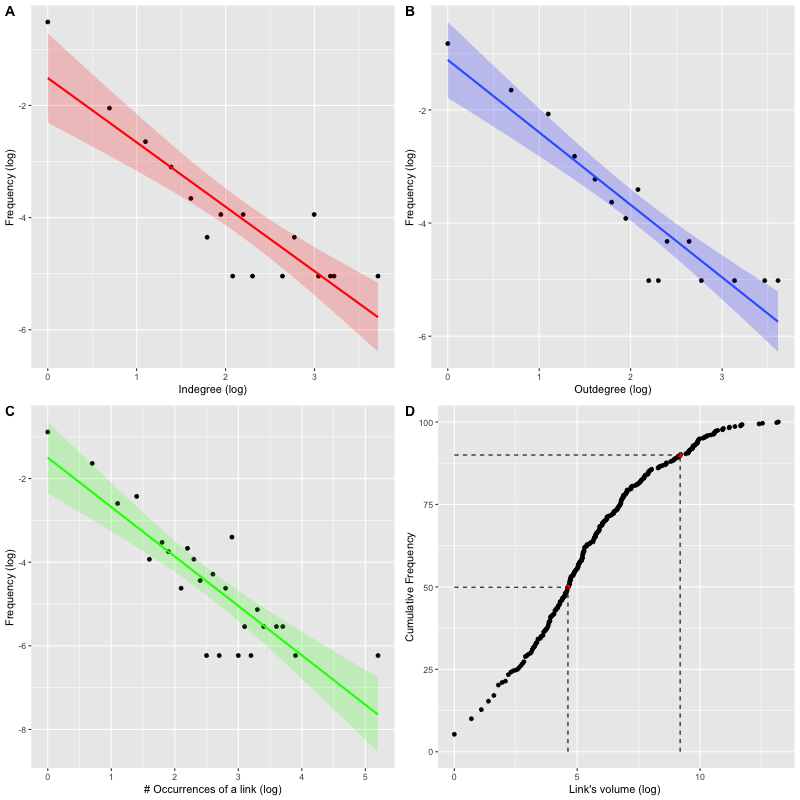

Supplement: S3 Fig — Indegree (A), outdegree (B) and frequency of the link (C) distribution in log-log scale and the link’s weight cumulative distribution in log scale (D). Shaded areas correspond to the results of linear regression, the dashed lines correspond to the 50th and 90th percentiles. (TIFF) [file ppat.1009397.s003.tiff]

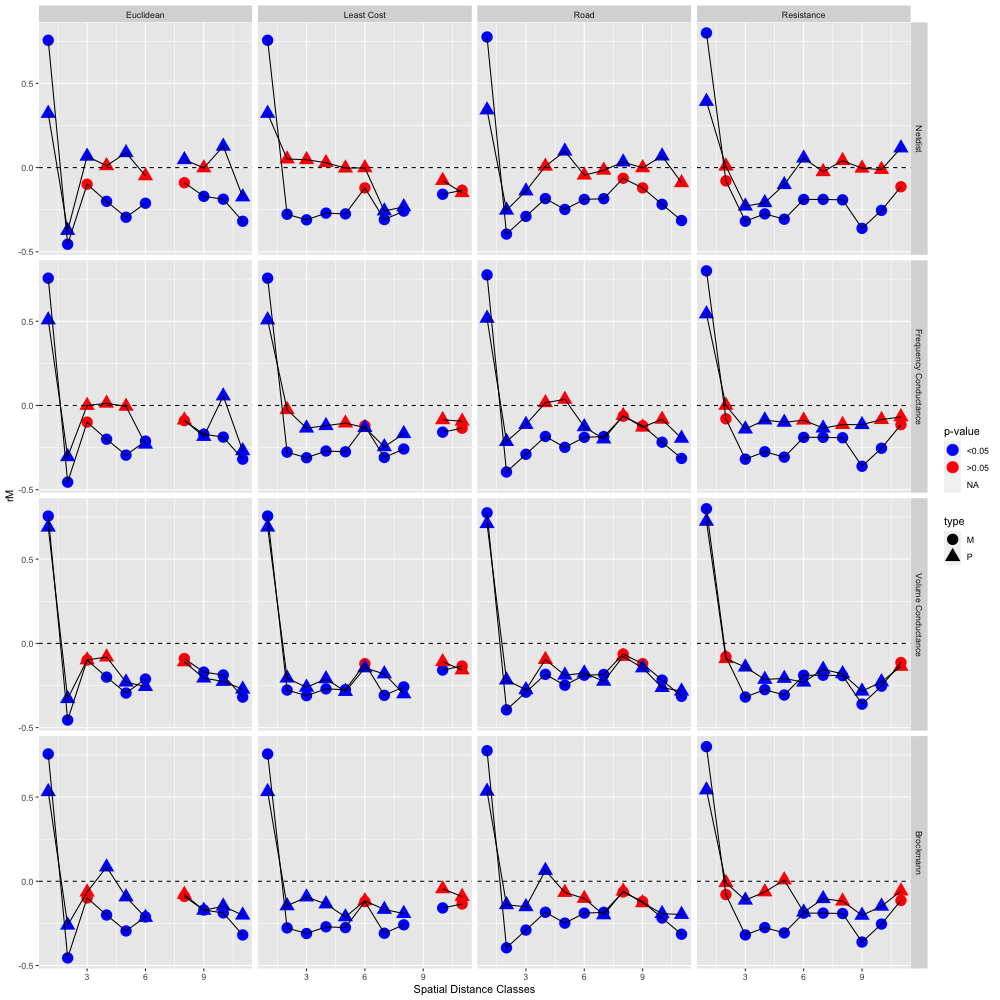

Supplement: S4 Fig — Mantel correlogram for all spatial distances considered (column), with (triangles) and without (circle) control by network distances (line). Color corresponds to significant and non-significant Mantel coefficients. (TIFF) [file ppat.1009397.s004.tiff]

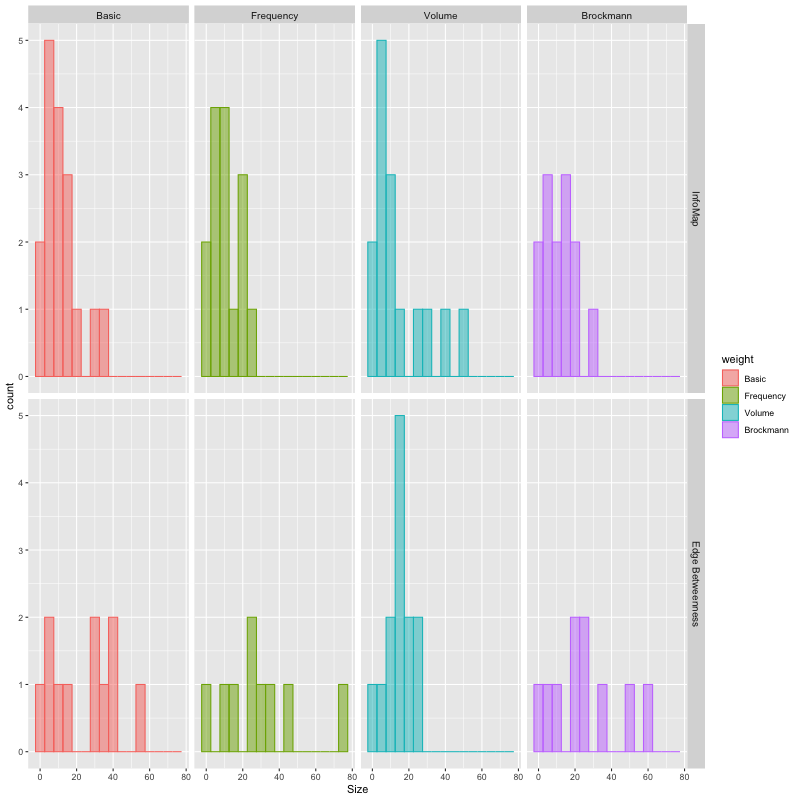

Supplement: S5 Fig — Community size distribution, based on the community detection algorithm used (row) and the link’s weight definition (column). (TIFF) [file ppat.1009397.s005.tiff]

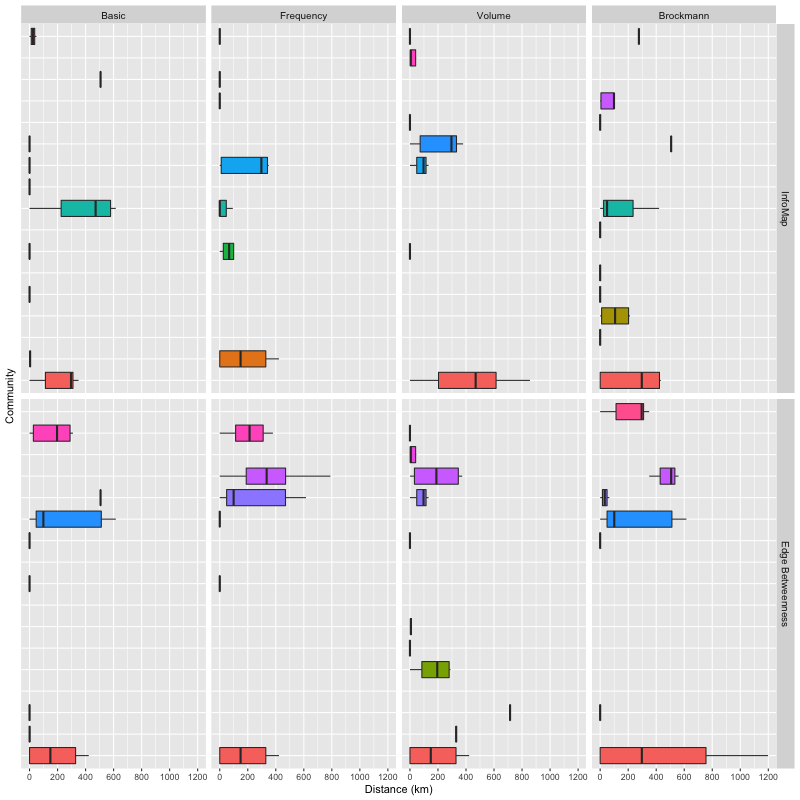

Supplement: S6 Fig — Community members’ distance distribution based on the community detection algorithm used (row) and the link’s weight definition (column). Colours corresponds to the community index. Each boxplot shows the distribution of geographical distance among locations belonging to the same community. (TIFF) [file ppat.1009397.s006.tiff]

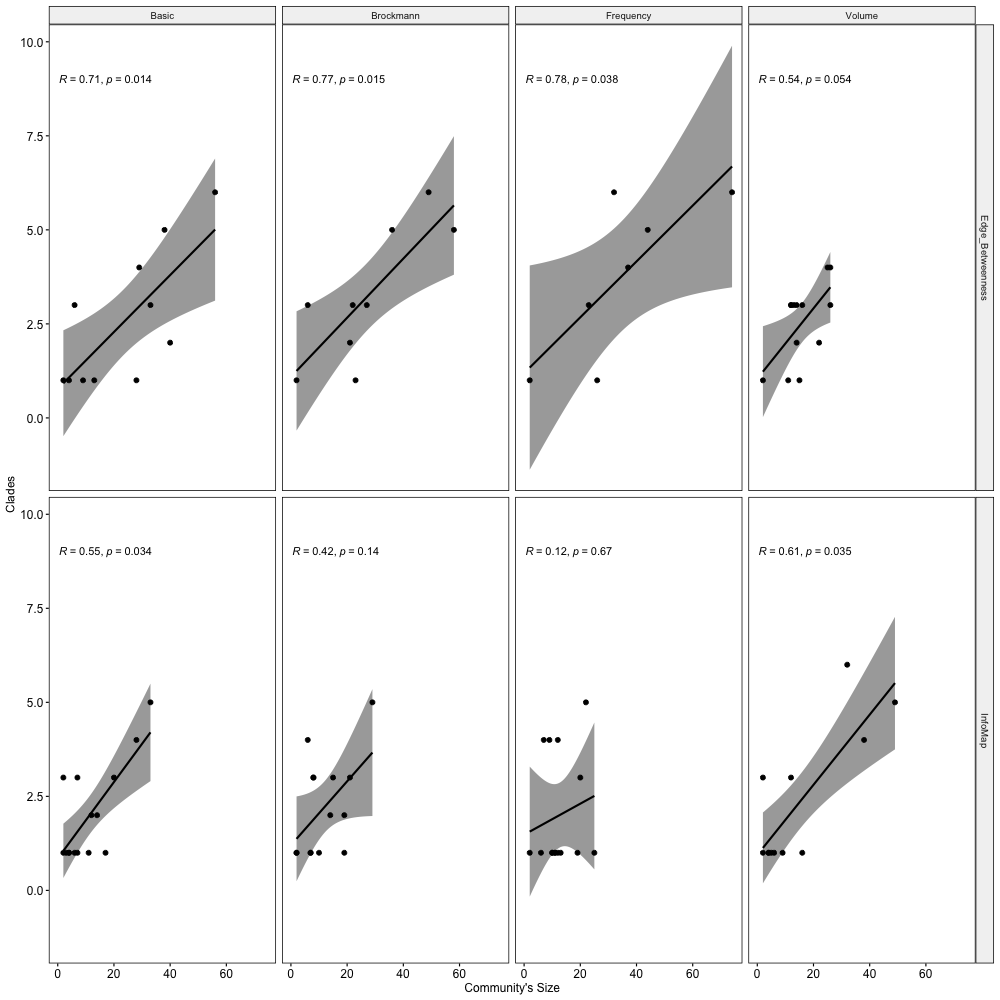

Supplement: S7 Fig — Correlation between community size and number of clades based on the community detection algorithm used (row) and the link’s weight definition (column). (TIFF) [file ppat.1009397.s007.tiff]
