# Supplementary material for: Combining viral genetic and animal mobility network data to unravel peste des petits ruminants transmission dynamics in West Africa
Source: PLoS Pathog. 2021 Mar 18;17(3):e1009397. doi: 10.1371/journal.ppat.1009397 (PMC8009415; doi:10.1371/journal.ppat.1009397)
Supplement: S1 Table — (DOCX) [file ppat.1009397.s008.docx]

**Table S1. List of samples collected and tested for Peste des Petits Ruminants infection.**

| **Region** | **Village/commune** | **N** | | **Year** | | **Lat** | | **Long** | |
| --- | --- | --- | --- | --- | --- | --- | --- | --- | --- |
| **Senegal** |  |  | |  | |  | |  | |
| Dakar | Dakar | 9/40 | | 2012-2013 | | 14,74054 | | -17,40752 | |
|  |  | 1/1 | | 2013 | | 14,74054 | | -17,40752 | |
| Fatick | Keur Moda | 6/66 | | 2012 | | 14,03252 | | -16,36285 | |
|  | Soum | 2/2 | | 2012 | | 14,08509 | | -16,47950 | |
| Kaolack | Ndiaffate | 2/5 | | 2012-2013 | | 14,08134 | | -16,21701 | |
|  | Mban Saman | 3/5 | | 2012 | | 14,13010 | | -16,23137 | |
|  | Ndiathiane | 2/10 | | 2012 | | 14,03859 | | -16,23034 | |
| Kedougou | Kedougou | 2/32 | | 2012-2013 | | 12.55126 | | -12.2020 | |
| Kolda | Commune | 1/1 | | 2014 | | 12,88333 | | -14,95 | |
|  | Bel air | 0/10 | | 2013 | | 12,89999 | | -14,95445 | |
|  | Pakour | 2/2 | | 2013 | | 12,75992 | | -13,96226 | |
|  | Médina Bourré | 0/5 | | 2013 | | 12,72499 | | -13,76277 | |
|  | Vélingara | 1/8 | | 2013 | | 13,15092 | | -14,10037 | |
|  | Gadapara | 1/5 | | 2013 | | 12,88891 | | -14,9499 | |
| Louga | Louga | 3/15 | | 2012 | | 15,60954 | | -16,23699 | |
|  | Linguère | 2/11 | 2012-2013 | | 15,21371 | | -15,16362 | |  |
|  | Sakal | 0/2 | | 2013 | | 15,82277 | | -16,23454 | |
| Matam | Sinthiou bamambé | 1/34 | | 2013 | | 15,6015 | | -13,3204 | |
|  | Ourosigui | 2/47 | | 2013 | | 15,36880 | | -13,13655 | |
|  | Ndendory | 2/55 | | 2013 | | 15,39316 | | -13,5287 | |
|  | Ouali diala | 2/39 | | 2013 | | 15.28444 | | -13.01333 | |
|  | Vindou Bosseable | 3/53 | | 2013 | | 15,30028 | | -12,93333 | |
|  | Gasambery | 1/49 | | 2013 | | 15,3976 | | -13,5287 | |
| Saint-Louis | Ross bétio | 0/17 | | 2013 | | 16,27686 | | -1614728 | |
|  | Ouro Islam | 0/4 | | 2013 | | 16,26616 | | -16,80728 | |
| Sédhiou | Koussy | 0/5 | | 2013 | | 12,87160 | | -15,60461 | |
|  | Sédhiou | 0/5 | | 2013 | | 12,70447 | | -15,55165 | |
| Tambacounda | Ndoga Babacar | 6/78 | | 2012-2013 | | 13,68643 | | -14,04269 | |
|  | Medina | 1/14 | | 2014 | | 13,70373 | | -13,7453 | |
|  | Saré Kali | 2/7 | | 2013 | | 13,69976 | | -13,60586 | |
| Thiès | Fissel | 1/11 | | 2010 | | 14,54378 | | -16,61355 | |
|  | Nguekhokh | 5/11 | | 2010 | | 14,52367 | | -17,01317 | |
|  | Ngairing | 10/10 | | 2010 | | 14,46006 | | -17,04131 | |
|  | Sâkh Mécké | 6/6 | | 2012 | | 14,93879 | | -16,49139 | |
|  | Gaye Mécké | 1/33 | | 2013 | | 15,09467 | | -16,48886 | |
|  | Baba Garage | 1/69 | | 2013 | | 14,944447 | | -16,66555 | |
|  | Mbour Joal | 2/61 | | 2013 | | 14,17462 | | -16,8390 | |
| **Guinea** |  |  | |  | |  | |  | |
|  | Dalaba | 2/2 | | 2013 | | 10,68684 | | -12,24903 | |
|  | Kaala | 1/1 | | 2013 | | 10,81657 | | -12,16663 | |
| **Mali** |  |  | |  | |  | |  | |
|  | Kolondieba | 6/20 | | 2014 | | 11.084 | | -6.899 | |
|  | Sagabari | 2/10 | | 2014 | | 12.586 | | -9.804 | |
|  | Sosorona | 1/1 | | 2014 | | 13.572 | | -5.989 | |
| **Mauritania** |  |  | |  | |  | |  | |
|  | Tarza Tignarmaik | 3/7 | | 2012 | | 17,51635 | | -17,31625 | |
|  |  |  | |  | |  | |  | |

N, number of PPR-positive samples/total samples collected (based on RT-PCR results); Lat, latitude; Long, longitude.
