# Supplementary material for: Combining viral genetic and animal mobility network data to unravel peste des petits ruminants transmission dynamics in West Africa
Source: PLoS Pathog. 2021 Mar 18;17(3):e1009397. doi: 10.1371/journal.ppat.1009397 (PMC8009415; doi:10.1371/journal.ppat.1009397)
Supplement: S2 Table — (DOCX) [file ppat.1009397.s009.docx]

**Table S2. List of PPRV nucleoprotein and hemagglutinin gene sequences obtained in this study**

| **Name** | **Species** | **Sample** | **Date** | ***(partial)* N** | **H** |
| --- | --- | --- | --- | --- | --- |
| **Senegal** |  |  |  |  |  |
| DakarP2 | goat | Os | 28/02/2012 | MT072450 | MT072500 |
| DakarP4 | goat | Os | 28/02/2012 | MT072463 | MT072513 |
| DakarP6 | goat | Os | 28/02/2012 | MT072451 | MT072501 |
| DakarP7 | goat | Os | 28/02/2012 | *MT072451* | - |
| Dakar7 | goat | Os | 01/03/2013 | *MT072452* | - |
| Dakar11° | goat | Os | 01/03/2013 | MT072452 | MT072512 |
| Dakar12 | goat | Os | 01/03/2013 | *MT072479* | - |
| Dakar18 | goat | Os | 01/03/2013 | *MT072479* | - |
| Dakar37 | sheep | Os | 01/03/2013 | MT072479 | MT072529 |
| KeurModa59 | goat | Os | 18/03/2012 | MT072448 | MT072498 |
| KeurModa60 | goat | Os | 18/03/2012 | *MT072448* | - |
| KeurModa61 | goat | Os | 18/03/2012 | *MT072448* | - |
| KeurModa62 | goat | Os | 18/03/2012 | *MT072448* | - |
| KeurModa01 | goat | Os | 30/04/2012 | *MT072448* | - |
| KeurModa02 | goat | Os | 30/04/2012 | *MT072448* | - |
| Soum01 | goat | Os | 08/03/2012 | MT072461 | MT072511 |
| Soum02 | goat | Os | 08/03/2012 | MT072494 | MT072544 |
| Ndiaffate01 | goat | Os | 15/03/2012 | MT072460 | MT072510 |
| Ndiaffate02 | goat | Os | 15/03/2012 | *MT072460* | - |
| MbanSaman01 | goat | Os | 17/03/2012 | MT072459 | MT072509 |
| MbanSaman03 | goat | Os | 17/03/2012 | *MT072459* | - |
| MbanSaman04 | goat | Os | 17/03/2012 | *MT072459* | - |
| Ndiathiane01 | goat | Os | 17/03/2012 | MT072462 | MT072512 |
| Ndiathiane06 | goat | Os | 17/03/2012 | *MT072462* | - |
| Kedougou1 | goat | Os | 08/03/2013 | MT072465 | MT072515 |
| Kedougou2 | goat | Os | 09/03/2013 | MT072466 | MT072516 |
| Kolda14 | goat | Os | 08/03/2013 | MT072473 | MT072523 |
| Gadapara1 | goat | Os | 06/03/2013 | *MT072473* | - |
| Pakour1 | goat | Os | 08/03/2013 | MT072475 | MT072525 |
| Pakour2 | goat | Os | 08/03/2013 | MT072476 | MT072526 |
| Velingara | goat | Os | 08/03/2013 | MT072474 | MT072524 |
| Louga2 | goat | Os | 25/04/2012 | MT072454 | MT072504 |
| SinthiouBamambe | goat | Os | 09/03/2013 | MT072455 | MT072505 |
| Ourosigui1 | goat | Os | 10/03/2013 | *MT072455* | - |
| Ourosigui4 | goat | Os | 10/03/2013 | MT072447 | MT072497 |
| Ndendory3 | goat | Os | 10/03/2013 | *MT072452* | - |
| Ndendory55 | goat | Os | 10/03/2013 | MT072470 | MT072520 |
| OualiDiala3 | goat | Os | 10/03/2013 | *MT072452* | - |
| OualiDiala4 | goat | Os | 10/03/2013 | *MT072452* | - |
| VindouBosseable1 | goat | Os | 10/03/2013 | *MT072452* | - |
| VindouBosseable 2 | goat | Os | 10/03/2013 | *MT072452* | - |
| VindouBosseable 3 | goat | Os | 10/03/2013 | *MT072452* | - |
| Gasambery1 | goat | Os | 10/03/2013 | MT072471 | MT072521 |
| NdogaBabacar1201 | goat | Os | 14/03/2012 | MT072464 | MT072514 |
| NdogaBabacar1214 | goat | Os | 14/03/2012 | *MT072464* | - |
| NdogaBabacar1301 | goat | Os | 08/02/2013 | *MT072464* | - |
| NdogaBabacar1302 | goat | Os | 08/02/2013 | *MT072464* | - |
| NdogaBabacar1303 | goat | Lg | 08/02/2013 | *MT072464* | - |
| NdogaBabacar1304 | goat | Lg | 08/02/2013 | MT072453 | MT072503 |
| Medina | goat | Os | 11/01/2014 | MT072472 | MT072522 |
| SareKali1 | goat | Lg | 08/03/2013 | MT072490 | MT072540 |
| SareKali2 | goat | Lg | 08/03/2013 | MT072491 | MT072541 |
| Kompentoum1 | goat | Os | 08/03/2013 | *MT072464* | - |
| Fissel11 | goat | Os | 25/04/2010 | MT072449 | MT072499 |
| Nguekhokh1 | goat | Os | 25/04/2010 | MT072483 | MT072533 |
| Nguekhokh2 | goat | Os | 25/04/2010 | MT072481 | MT072531 |
| Nguekhokh3 | goat | Os | 25/04/2010 | MT072482 | MT072532 |
| Nguekhokh4 | goat | Os | 25/04/2010 | *MT072482* | - |
| Nguekhokh5 | goat | Os | 25/04/2010 | *MT072482* | - |
| Ngairing1 | goat | Os | 28/04/2010 | MT072489 | MT072539 |
| Ngairing2 | goat | Os | 28/04/2010 | *MT072489* | - |
| Ngairing3 | goat | Os | 28/04/2010 | *MT072489* | - |
| Ngairing4 | goat | Os | 28/04/2010 | *MT072489* | - |
| Ngairing5 | goat | Os | 28/04/2010 | *MT072489* | - |
| Ngairing6 | goat | Os | 28/04/2010 | *MT072489* | - |
| Ngairing7 | goat | Os | 28/04/2010 | *MT072489* | - |
| Ngairing8 | goat | Os | 28/04/2010 | *MT072489* | - |
| Ngairing9 | goat | Os | 28/04/2010 | *MT072489* | - |
| Ngairing10 | goat | Os | 28/04/2010 | *MT072489* | - |
| SakhMecke1 | goat | Os | 21/05/2012 | MT072458 | MT072498 |
| SakhMecke2 | goat | Os | 21/05/2012 | *MT072458* | - |
| SakhMecke3 | goat | Os | 21/05/2012 | MT072492 | MT072542 |
| SakhMecke4 | goat | Os | 21/05/2012 | *MT072458* | - |
| SakhMecke5 | goat | Os | 21/05/2012 | *MT072458* | - |
| SakhMecke6 | goat | Os | 21/05/2012 | *MT072458* | - |
| GayeMecke1 | goat | Lg | 05/01/2013 | MT072478 | MT072528 |
| BabaGarage1 | goat | Os | 10/02/2013 | *MT072458* | - |
| MbourJoal1 | goat | Lg | 05/01/2013 | MT072445 | MT072495 |
| MbourJoal2 | goat | Lg | 05/01/2013 | MT072446 | MT072496 |
| **Guinea** |  |  |  |  |  |
| Dalaba1 | goat | Lg | 01/02/2013 | MT072467 | MT072517 |
| Dalaba2 | goat | Lg | 01/02/2013 | MT072469 | MT072519 |
| Kaala | goat | Lg | 01/02/2013 | MT072468 | MT072518 |
| **Mali** |  |  |  |  |  |
| Kolondieba1 | goat | Os | 27/07/2013 | MT072484 | MT072534 |
| Kolondieba2 | goat | Os | 27/07/2013 | MT072485 | MT072535 |
| Kolondieba3 | goat | Os | 27/07/2013 | MT072486 | MT072536 |
| Kolondieba4 | goat | Os | 27/07/2013 | MT072488 | MT072538 |
| Kolondieba6 | goat | Os | 27/07/2013 | MT072493 | MT072543 |
| Kolondieba18 | goat | Os | 27/07/2013 | MT072487 | MT072537 |
| Sagabary4 | goat | Os | 10/07/2013 | MT072480 | MT072530 |
| Sagabary10 | goat | Os | 10/07/2013 | MT072477 | MT072527 |
| Sosorona1* | goat | Os | 10/07/2013 | *MT072545* | - |
| **Mauritania** |  |  |  |  |  |
| TarzaTignarmaik1 | Sheep | Os | 04/01/2012 | MT072456 | MT072506 |
| TarzaTignarmaik4 | Sheep | Os | 04/01/2012 | MT072457 | MT072497 |
| TarzaTignarmaik7 | Sheep | Os | 04/01/2012 | *MT072457* |  |
|  |  |  |  |  |  |

Lg, lung; Os, ocular swab; Accession number, GenBank accession number. N, complete nucleoprotein gene sequences obtained. The accession number is in italics if only a partial sequence of 255 bp was obtained. H, complete hemagglutinin gene sequences obtained. Accession numbers are repeated when the same sequence was obtained from several locations. (*) Only sample of PPRV lineage I, whereas all other samples belonged to lineage II (see results in Fig. S1). (°) Sample with full genome already sequenced in earlier publication (Genbank accession number: KM212177).
