# Supplementary material for: Combining viral genetic and animal mobility network data to unravel peste des petits ruminants transmission dynamics in West Africa
Source: PLoS Pathog. 2021 Mar 18;17(3):e1009397. doi: 10.1371/journal.ppat.1009397 (PMC8009415; doi:10.1371/journal.ppat.1009397)
Supplement: S3 Table — (DOCX) [file ppat.1009397.s010.docx]

**Table S3. Correlation coefficients between genetic distance and spatial and network measures.** On the left, spatial distances based on the geographical position of the strains. On the right, network-related distances evaluated considering the distance in terms of links crossed in the unweighted (Netdist) or weighted cases. Resistance and conductance measurements were made using CircuitScape.

| **Spatial distances** | | **Network-related distances** | |  |
| --- | --- | --- | --- | --- |
| Euclidean | 0.71 | Netdist | 0.79 | |
| Road | 0.72 | Frequency Conductance | 0.72 | |
| Least cost | 0.73 | Volume Conductance | 0.53 | |
| Friction/Resistance | 0.76 | Brockmann Resistance | 0.73 | |

All correlation coefficients were highly significant (p-value < 0.001; permutation test)
