# Supplementary material for: Combining viral genetic and animal mobility network data to unravel peste des petits ruminants transmission dynamics in West Africa
Source: PLoS Pathog. 2021 Mar 18;17(3):e1009397. doi: 10.1371/journal.ppat.1009397 (PMC8009415; doi:10.1371/journal.ppat.1009397)
Supplement: S4 Table — (DOCX) [file ppat.1009397.s011.docx]

**Table S4. Partial Mantel tests of correlation coefficients between genetic distance and spatial and network measures.** Each entry in the table corresponds to the result of a partial Mantel test between PPRV genetic distance and different network and spatial distances. Each test for network-related distances was performed using different spatial distances as control variables (upper part of the table). The same was done for tests with distance distances controlled with different network-related distances (bottom part of the table).

|  | **Control Variable (spatial distances)** | | | | |
| --- | --- | --- | --- | --- | --- |
| **Regressor (Network-related distances)** |  | Euclidean | Road | Least Cost | Friction Resistance |
|  | Netdist | 0.50 | 0.56 | 0.59 | 0.35 |
|  | Frequency Conductance | 0.50 | 0.49 | 0.53 | 0.36 |
|  | Volume Conductance | 0.47 | 0.47 | 0.38 | 0.34 |
|  | Brockmann Resistance | 0.56 | 0.56 | 0.53 | 0.43 |
|  | **Control Variable (Network related distances)** | | | | |
| **Regressor (Spatial distances)** |  | Netdist | Frequency Conductance | Volume Conductance | Brockmann Resistance |
|  | Euclidean | 0.096 | 0.41 | 0.64 | 0.46 |
|  | Road | 0.08 | 0.41 | 0.64 | 0.47 |
|  | Least Cost | 0.37 | 0.46 | 0.60 | 0.44 |
|  | Friction/Resistance | 0.13 | 0.41 | 0.66 | 0.47 |

All correlation coefficients were highly significant (p-value < 0.001; permutation test)
