# Supplementary material for: Combining viral genetic and animal mobility network data to unravel peste des petits ruminants transmission dynamics in West Africa
Source: PLoS Pathog. 2021 Mar 18;17(3):e1009397. doi: 10.1371/journal.ppat.1009397 (PMC8009415; doi:10.1371/journal.ppat.1009397)
Supplement: S5 Table — (DOCX) [file ppat.1009397.s012.docx]

**Table S5. Results of the Multiple Regression on distance matrices analyses (MRM).** Each line corresponds to the coefficients of MRM using a network and a spatial distance. Only results where at least one coefficient is significant have been included.

| **Geo dist** | **Coeff Geo** | **Net dist** | **Coef Net** | **Intercept** | **R2** | **Dominant** |
| --- | --- | --- | --- | --- | --- | --- |
| **Short distance Class ≤ 158 km (59 pairs)** | | | | | | |
| Euclidean | -0.0026*** | Brockmann | 5.8E-05** | 9.5E-08 | 0.72*** | Geographical |
| Euclidean | -0.0027*** | Conductanceweight | 0.0006* | 5.6E-07 | 0.72*** | Geographical |
| Euclidean | -0.0026*** | Conductancefreq | 0.0004** | 2.0E-07 | 0.72*** | Geographical |
| LeastCost | -0.0024*** | Brockmann | 5.3E-05* | 1.1E-07 | 0.72*** | Geographical |
| LeastCost | -0.0025*** | Conductanceweight | 0.0005* | 5.8E-07 | 0.72*** | Geographical |
| LeastCost | -0.0024*** | Conductancefreq | 0.0004* | 1.4E-07 | 0.72*** | Geographical |
| Resist | -0.0019*** | Netdist | -0.0004* | 1.9E-06 | 0.65*** | Geographical |
| Resist | -0.0019*** | Brockmann | -0.0004* | 1.9E-06 | 0.65*** | Geographical |
| Resist | -0.0019*** | Conductanceweight | -0.0150* | 1.9E-06 | 0.65*** | Network |
| Resist | -0.0019*** | Conductancefreq | -0.0022* | 1.9E-06 | 0.65*** | Network |
|  |  |  |  |  |  |  |
| **Long distance Class > 158 km (59 pairs)** | | | | | | |
| Euclidean | 0.0006*** | Netdist | 0.0008*** | 1.3E-05 | 0.82*** | Network |
| Euclidean | 0.0013*** | Brockmann | 0.0004*** | 4.0E-05 | 0.80*** | Geographical |
| Euclidean | 0.0018*** | Conductanceweight | 0.0028*** | 7.3E-05 | 0.71*** | Network |
| Euclidean | 0.0012*** | Conductancefreq | 0.0016*** | 3.9E-05 | 0.76*** | Network |
| LeastCost | 0.0009*** | Netdist | 0.0007*** | 1.1E-05 | 0.83*** | Geographical |
| LeastCost | 0.0007*** | Brockmannabove | 0.0004*** | 8.7E-05 | 0.70*** | Geographical |
| LeastCost | 0.0012*** | Conductanceweight | 0.0029*** | 0.0002 | 0.48*** | Network |
| LeastCost | 0.0008*** | Conductancefreq | 0.0023*** | 8.6E-05 | 0.68*** | Network |
| Resist | -0.0006*** | Netdist | 0.0009*** | 4.1E-05 | 0.77*** | Network |
| Resist | 0.0007*** | Brockmann | 0.0004*** | 0.0001 | 0.68*** | Geographical |
| Resist | 0.0011*** | Conductanceweight | 0.0032*** | 0.0002 | 0.44*** | Network |
| Resist | 0.0004*** | Conductancefreq | 0.0023*** | 0.0001 | 0.64*** | Network |
| Road | 0.0001 | Netdist | 0.0007*** | 3.4E-05 | 0.75*** | Network |
| Road | 0.0010*** | Brockmann | 0.0003*** | 7.5E-05 | 0.71*** | Geographical |
| Road | 0.0015*** | Conductanceweight | 0.0031*** | 0.0002 | 0.53*** | Network |
| Road | 0.0009*** | Conductancefreq | 0.0019*** | 7.2E-05 | 0.68*** | Network |

Geo Dist, different types of geographic distances; Coeff Geo, correlation coefficient between geographic and genetic distances; Net dist, different types of network-related distances; Coef Net, correlation coefficient between network-related and genetic distances; intercept, value of the intercept in the regression analysis; R2, measure of goodness-of-fit of the regression models; Dominant, indicate the distance variable (geographic vs network-related) of most importance in the regression analysis. Significance of coefficients was evaluated with permutation tests (p < 0.05*, <0.01**, <0.001***).
