# Supplementary material for: Combining viral genetic and animal mobility network data to unravel peste des petits ruminants transmission dynamics in West Africa
Source: PLoS Pathog. 2021 Mar 18;17(3):e1009397. doi: 10.1371/journal.ppat.1009397 (PMC8009415; doi:10.1371/journal.ppat.1009397)
Supplement: S6 Table — (DOCX) [file ppat.1009397.s013.docx]

**Table S6.** **Number of sequences available around a network node and type of node.**

| **Node** | **#sequences** | **#clades** | **Type** |
| --- | --- | --- | --- |
| Dande | 2 | 1 | Monoclade |
| Joal | 2 | 1 | Monoclade |
| Kaolack | 2 | 1 | Monoclade |
| Keur Sam Pendel | 1 | 1 | Monoclade |
| Khombole | 1 | 1 | Monoclade |
| Kolda | 1 | 1 | Monoclade |
| Mbam | 2 | 2 | Hotspot |
| Mbour | 3 | 1 | Monoclade |
| Meouane | 2 | 2 | Hotspot |
| Ndoffane | 1 | 1 | Monoclade |
| Niono | 1 | 1 | Monoclade |
| Ogo | 2 | 1 | Monoclade |
| Ouad Naga | 3 | 1 | Monoclade |
| Ourossogui | 1 | 1 | Monoclade |
| Paroumba | 2 | 1 | Monoclade |
| Pikine | 6 | 3 | Hotspot |
| Saraya | 1 | 1 | Monoclade |
| Sinthiou Bamambe | 1 | 1 | Monoclade |
| Souleymaniya | 3 | 1 | Monoclade |
| Tambacounda | 1 | 1 | Monoclade |
| Thiara | 2 | 2 | Hotspot |
| Velingara | 1 | 1 | Monoclade |

#sequences and #clades indicate, respectively, the number of sequences collected in the area and number of different genetic clades they belong to. Type indicates if the village is classified as a Monoclade (all the strains belong to the same clade) or Hotspot (strains belong to different clades). Only sequences belonging to well-defined clades (clades 1 to 7) were considered.
