# Supplementary material for: Combining viral genetic and animal mobility network data to unravel peste des petits ruminants transmission dynamics in West Africa
Source: PLoS Pathog. 2021 Mar 18;17(3):e1009397. doi: 10.1371/journal.ppat.1009397 (PMC8009415; doi:10.1371/journal.ppat.1009397)
Supplement: S7 Table — (DOCX) [file ppat.1009397.s014.docx]

**Table S7.** **Average values of network characteristics in hotspot and monoclade nodes**

|  | **Sequences** | **Indegree** | **Outdegree** | **InFrequency** | **Inweight** | **Outweight** | **Centrality** | **Betweenness** |
| --- | --- | --- | --- | --- | --- | --- | --- | --- |
| Monoclade | 1.6 | 2.9 | 1.4 | 5.8 | 890 | 7793 | 0.12 | 0.04 |
|  | (0.4) | (2.5) | (1.25) | (4.6) | (1160) | (10600) | (0.65) | (0.02) |
| Hotspot | 3 | 11 | 0 | 2.8 | 7508 | 0 | 0.20 | 0.22 |
|  | (1) | (10) | (0) | (1.8) | (7490) | (0) | (0.17) | (0.1) |
| Homophily | | | | | | | | |
|  | InfoMap | | | | Edge_Betweenness | | | |
|  | Basic | Frequency | Volume | Brockmann | Basic | Frequency | Volume | Brockmann |
| Monoclade | 0.72 | 0.71 | 0.68 | 0.73 | 0.82 | 0.88 | 0.71 | 0.79 |
|  | (0.29) | (0.26) | (0.27) | (0.25) | (0.23) | (0.18) | (0.25) | (0.23) |
| Hotspot | 0.85 | 0.80 | 0.87 | 0.83 | 0.90 | 0.93 | 0.80 | 0.88 |
|  | (0.30) | (0.40) | (0.26) | (0.33) | (0.21) | (0.15) | (0.40) | (0.23) |
| Homophily strength (frequency of exchanges) | | | | | | | | |
|  | InfoMap | | | | Edge_Betweenness | | | |
|  | Basic | Frequency | Volume | Brockmann | Basic | Frequency | Volume | Brockmann |
| Monoclade | 0.84 | 1.11 | 1.08 | 0.82 | 0.95 | 0.95 | 1.00 | 0.98 |
|  | (0.30) | (0.29) | (0.32) | (0.27) | (0.17) | (0.17) | (0.28) | (0.28) |
| Hotspot | 0.83 | 1.48 | 1.15 | 0.79 | 0.82 | 0.85 | 0.80 | 0.82 |
|  | (0.33) | (0.96) | (0.31) | (0.41) | (0.36) | (0.30) | (0.48) | (0.36) |
| Homophily strength (volume of exchanges) | | | | | | | | |
|  | InfoMap | | | | Edge_Betweenness | | | |
|  | Basic | Frequency | Volume | Brockmann | Basic | Frequency | Volume | Brockmann |
| Monoclade | 0.63 | 0.68 | 0.81 | 0.58 | 0.79 | 0.88 | 0.73 | 0.75 |
|  | (0.40) | (0.37) | (0.23) | (0.40) | (0.26) | (0.20) | (0.22) | (0.31) |
| Hotspot | 0.84 | 0.82 | 0.97 | 0.76 | 0.85 | 0.87 | 0.75 | 0.78 |
|  | (0.30) | (0.36) | (0.05) | (0.48) | (0.29) | (0.27) | (0.23) | (0.23) |

Values in parentheses represent standard deviation*.* Homophily, i.e. the tendency to create connections with members of the same community, is estimated for each community partition, and for different types of strength of connection. All these characteristics are considered as possible “predictors” to identify hotspot locations, i.e. factors that could justify the presence of strains of more than a single clade.
