# Supplementary material for: Combining viral genetic and animal mobility network data to unravel peste des petits ruminants transmission dynamics in West Africa
Source: PLoS Pathog. 2021 Mar 18;17(3):e1009397. doi: 10.1371/journal.ppat.1009397 (PMC8009415; doi:10.1371/journal.ppat.1009397)
Supplement: S8 Table — (DOCX) [file ppat.1009397.s015.docx]

**Table S8.** **Results of Linear Discriminant Analysis**.

|  | InfoMap | | | | | Edge_Betweenness | | | |
| --- | --- | --- | --- | --- | --- | --- | --- | --- | --- |
|  | Basic | Frequency | Volume | Brockmann | | Basic | Frequency | Volume | Brockmann |
| Sequences | -3.8E-02 | 6.5E-04 | 2.9E-02 | -5.2E-02 | | -2.2E-02 | 4.9E-02 | -1.5E-01 | -1.2E-01 |
| Indegree | -0.2 | -0.2 | -0.1 | -0.2 | | -0.1 | 0.01 | -0.2 | -0.2 |
| Outdegree | -0.01 | -0.03 | -0.04 | -0.04 | | 0.01 | 0.001 | -0.01 | 0.01 |
| InFrequency | -3.7E-04 | -4.0E-04 | -3.5E-04 | -2.7E-04 | | -1.9E-04 | -2.4E-04 | -6.6E-05 | -1.6E-04 |
| Inweight | 2.1E-06 | 2.4E-05 | 1.4E-05 | 1.4E-05 | | -2.7E-05 | -2.4E-05 | -9.3E-06 | -2.3E-05 |
| Outweight | 2.7E-03 | 2.1E-03 | 2.2E-03 | 2.3E-03 | | 2.4E-03 | 2.4E-03 | 1.7E-03 | 2.3E-03 |
| Beweenness | -5.33 | -0.01 | -6.12 | -4.0 | | -10.9 | -14.6 | -0.8 | -5.5 |
| Eigenvector Centrality | -3.8E-02 | 6.5E-04 | 2.9E-02 | -5.2E-02 | | -2.2E-02 | 4.9E-02 | -1.5E-01 | -1.2E-01 |
| Homophily | 2.1 | 3.8 | 2.3 | | 2.4 | 0.7 | -1.1 | 2.7 | 2.1 |

All values obtained were not significant (Kruskal -Wallis test, p-values > 0.05)
