# Supplementary material for: Combining viral genetic and animal mobility network data to unravel peste des petits ruminants transmission dynamics in West Africa
Source: PLoS Pathog. 2021 Mar 18;17(3):e1009397. doi: 10.1371/journal.ppat.1009397 (PMC8009415; doi:10.1371/journal.ppat.1009397)
Supplement: S9 Table — (DOCX) [file ppat.1009397.s016.docx]

**Table S9.** **Description of primers used to sequence the complete PPRV nucleoprotein and hemagglutinin genes**

| **Primer name** | **Genome position** | **Sequence (5’-3’)** |
| --- | --- | --- |
| **Nucleoprotein** |  |  |
| Nleader1 | 1>21 | ACCAGACAAAGCTGGGTAAGG |
| NP63f | 315>334 | ACCGGCGTGATGATCAGCAT |
| NP65f | 768>786 | AACAGGATTGCAGAAGATC |
| NP3f | 1232>1255 | GTCTCGGAAATCGCCTCACAGACT |
| PN340r | 367<389 | ATCATCTGTGATCCGCTGTATCA |
| PN65r | 768<786 | GATCTTCTGCAATCCTGTT |
| PN140r | 1395<1414 | TCTTTGGTCGCTGGTGTAGG |
| NP4r | 1560<1583 | CCTCCTCCTGGTCCTCCAGAATCT |
| PN1750r | 1745<1764 | GCTTGGACCTGGGTCCTAAG |
| **Hemagluttinin** |  |  |
| F25F | 7218>7238 | CGACACAAGCAGTCAGAGGACG |
| 2HPPRf | 7799>7820 | TGAACCCCCCTGAGAGAGTCA |
| 3HPPRf | 8298>8317 | AACCTAGCTGGGCCTACTCT |
| 4HPPRf | 8751>8770 | GCAGAGGTTATGCCGCACAT |
| 01HPPRr | 7840<7859 | TCGCAACCTTTCTGACCTGT |
| 02HPPRr | 8420<8842 | GGCAAGACACTGTATAGTTCG |
| 03HPPRr | 8823<8842 | TCATCAATCCTGCGGGACAA |
| 04HPPRr | 9269<9288 | TGAGTCCATGGCTCCTACAAC |

For both genes, forward primers are listed first, then reverse primers.
