# Supplementary material for: Combining viral genetic and animal mobility network data to unravel peste des petits ruminants transmission dynamics in West Africa
Source: PLoS Pathog. 2021 Mar 18;17(3):e1009397. doi: 10.1371/journal.ppat.1009397 (PMC8009415; doi:10.1371/journal.ppat.1009397)
Supplement: Attachment — Submitted filename: Response to reviewer-final.docx [file ppat.1009397.s022.docx]

Reviewer #1:

- *The authors identified six clades from their 50 PPRV sequences. However, on inspection of the phylogeny in Figure 1, these clades appear to genetically very similar. Therefore, it is unclear if these clades truly represent distinct lineages or they are just one single lineage (lineage II), which have been widely distributed. As this result/observation is crucial for some of the downstream analyses, I think the authors need to provide better justification/support for the six identified clades (e.g., by genetic distance or time to most recent common ancestor). There are methods for identifying transmission clusters using phylogenetics, particularly in HIV-1 and HCV epidemiology. Therefore, I think it would be worthwhile looking at these approaches (e.g.,* [*https://academic.oup.com/ve/article/5/2/vez039/5584304*](https://academic.oup.com/ve/article/5/2/vez039/5584304)*,* [*https://bmcbioinformatics.biomedcentral.com/articles/10.1186/1471-2105-14-317*](https://bmcbioinformatics.biomedcentral.com/articles/10.1186/1471-2105-14-317)*) when identifying transmission clusters of PPRV.*

Answer: The phylogenetic analyses performed in our study clearly identified genetic clusters within the lineage II, with strong statistical support obtained using two different inference methods. Pairwise genetic distance separating samples within clusters (mean distance: 0.30%) was significantly lower than sequence distance between samples from different clusters (mean distance: 0.52%; Wilcoxon test, W=10823, *p* < 0.0001). Still, we followed the reviewer’s suggestion to implement a method for identification of transmission clusters. We used ClusterPicker v1.2, following instructions of the developers, and obtained the same clusters. These additional analyses (genetic distance comparison and cluster identification software) provide further support that the clades identified can be considered as transmission clusters and can be used as such in downstream analyses.

The additional analyses (genetic distance comparison and cluster identification software) have been included in the text in the result section (L16-176) and method section (L485-486), and with a new Figure S2 in supplementary materials.

- *The results of the phylogenetic analyses need to be better explained in the main text. How many sequences were found per clade? What was their phylogenetic support? How was the main alignment curated? Did you use Genbank to include complete N and H PPRV sequences from relevant countries? How many sequences were included in the final alignment and how many new sequences did your study contribute? I was left wanting more detail about the analysis.*

Answer: We added information in the results and the methods sections, as requested. More precisely: the number of sequences per clade (L170-176), phylogenetic support (L170), method of phylogenetic analysis and alignment, and dataset used (L469-479, new supplementary material SM4).

- *Given it is a central result, I would like to see the correlation of genetic distance against spatial and network-related distances as a main figure, in addition to the statistical test. As there are only a limited number of sequences analysed in this study, I found it difficult to ascertain from the current analysis how many data points contribution in this correlation analysis. Likewise, I would also like to see the correlation plots for community size and the number of clades observed in these communities.*

Answer: New correlation analyses have been performed to answer comments below. The main result of one these analyses (Mantel correlogram) is now presented in the new Figure 4, complemented by a new Figure S4. Also, as suggested by the reviewer, we added a figure S7 for the correlation community size/number of strains has been included in the Supplementary material.

- *it would be helpful to have a short description/summary of the main methods used to infer networks/communities in the main text. It might be worthwhile to include a figure to illustrate what the authors mean by communities, and how related this information to virus genetic data to identify hotspots of transmission.*

Answer: The concept of communities in our study has been added in the introduction (L118-129) along with a new illustration to help readers (Figure 1). Description of the community detection method and the algorithm used also has been added in the Material and methods section (L567-593).

- *The results presented in Figure 3 is difficult to interpret, especially as someone with limited expertise in network analyses. The summary figure as suggested in the previous point might help address some of these concerns. However, I am still unclear what the colour labels for community in this figure correspond to. I appreciate panels A and B are showing results from the two different methods, but it is unclear if these results are showing a similar pattern or not.*

Answer: A summary Figure 1 has been added in the introduction along with some additional explanations (see above). In addition, several sentences have been added to legends of Figures 3 and 4 (now 4 and 5). The main goal of these figures is to show the spatial distribution of communities depending on the particular algorithm used and criteria. Each polygon, centred around network’s node, is coloured based on the community is part of. We are showing only communities where at least one strain has been found. The legend shows for each colour the community identity (label): each label corresponds to a different community. Since partition varies depending on algorithm used, the community structure is in principle different from one partition to another, therefore community identity in 2 different plots corresponds to different communities and there is no relation between them. Figures should help reader identifying if spatial structures are present, keeping in mind that structure can change from one community partition to another.

- *The cyan shading in Figure 3 is very jarring, especially when you have similarly coloured circles overlayed on top. Is this colour scheme necessary?*

Answer: The figure has been modified (number in revised manuscript = Figure 3).

- *Why was the mobility data from only 2014 selected and analysed. If this is due to availability of data, please clarify. If not, does the main findings hold when mobility data from other years are analysed instead?*

Answer: Mobility data have been collected in 2013, 2014 and 2015. However, very few and incomplete data were collected in 2013. Also, the 2015 data did not cover all the year, in particular few data were collected around the Tabaski period, during which most of the largest movements are concentrated. A paragraph has been added at L497-501.

- *Looking at the fasta files and Table S2, there appears to be 50 sequences that were generated in this study (not 49 as indicated in the main text), which includes 36 sequences from Senegal (not 39 as indicated in the main text).*

Answer: The number of sequences has been corrected in the text (L167 and 325).

- *Please provide a brief description of quadratic assignment procedure.*

Answer: Due to the non-normality of the output variable (see comments below), we decided to use Mantel and partial mantel test, Multiple Regression on distance matrices (MRM) method and Mantel correlograms to assess correlations between distances instead of QAP procedure in this revised manuscript. These methods are now described in the material and methods section (L553-566).

- *Figure 3 – axes are labelled simply ‘x’ and ‘y’. To be honest, the long/lat axes could be removed in this Figure as well as from Figure 4.*

Answer: Axes correspond to latitude and longitude. We believe that it is informative for the reader to keep the latitude/longitude labelling in these two figures.

Reviewer #2:

- *Evolution rate is very very low in PPRV as reported in many recent studies and main changes are seen in P gene. Therefore it is not convincing that each clade can be considered as part of different transmission networks. Analysis of full genome may be helpful, however as said above due to low evolutionary rate this may also be difficult.*

Answer: See answer to question 1 of reviewer #1

Reviewer #3:

- *L218-219: The authors write that genetic distance was more correlated to network distances than geographic distances. However, Table S2 shows that all distance with the exception of volume conductance ( a network distance), has similar correlations (between 0.72-0.79). So this statement is not supported by the data presented. This carries over to the discussion (L303-305)*

Answer: New correlation analyses have been performed to take into account the fact that distance data was not normally distribute and relationships between distances were not linear (see other comments). Mantel and partial Mantel tests showed that there was a strong correlation between virus genetic distance and both geographical and network-related distances, even after correction for interactions. However, results of Mantel correlograms and Multiple regression analyses suggest that the correlation between genetic and geographical distance is strong only among sites separated by relatively short distances (<100-150km), but that network connectivity best explained genetic distance patterns for larger distances. Such pattern supports the importance of animal mobility in PPRV transmission, as connections between markets separated by long distances depend on fast commercial transport that can rapidly move PPRV across the region. For shorter distances, mainly local movement of small ruminants would result in slower diffusion the virus.

The text in methods (L553-566), results (235-257), and discussion (340-349) sections has been changed to include these new analyses, with new figure 4, Tables S3-S5.

- *L224. Its unclear to me that MR-QAP is the appropriate approach for these data. MR-QAP is based on ordinary least squares, so the distribution of the outcome (genetic distance) should be someone normal. It would be beneficial for the authors to present this information. If the netlm assumptions are violated, it might be possible to dichomotmize the outcome and use netlogit. Also, which network and geographic distances were included in the regression. All of them? Are these variables co-linear, and how might that impact the outcome? The coefficient values are extremely small, which suggests that the x-variables were large. It might be beneficial to standardize these variables, or potentially put them on a log scale. Alternatively, consider created three levels.*

Answer: Indeed, to answer the reviewer’s comment, we performed a Shapiro-Wilk test and found that the genetic distance was not normally distributed. Therefore, we decided to change method to assess the role of the different distances in explaining the genetic distance. See responses to above comments.

- *L230. Here, the authors state that communities cluster spatially, but later in the paragraph they state there is no clear spatial pattern. Related to this, Figures 3 and 4 show that there is a great deal of variability in the community assignments, which begs the question if the community structure is somewhat weak and therefore inconsistent. It may help to report modularity scores.*

Answer: For all the community partition, the modularity is in the range 0.47-0.6, well above 0.3, thus indicating a good “partition” in communities. We used Clark Evans and join count tests to assess the presence of spatial pattern. Both the Clark Evans test and the join count test showed that there is a certain degree of spatial clustering for communities. In addition, we used the Rand Index to evaluate the similarity between communities is based on. Results showed that similarity is high. Looking at figures 5 and 6, we observe that communities show a high degree of spatial clustering in areas far from the main consumption centres as Dakar. These could be related to the production hierarchy. In the remote areas, movements are concentrated towards certain markets due to presence of stockists/traders.

The text in the methods (L526-532), results (219-223/258-273) and discussion sections (L356-359) have been modified to include further details on the community clustering and similarity analyses, and their possible interpretation.

- *L248. The word “risk” comes across oddly here. My understanding is that the authors aimed to determine if any two sequences were more likely to be in the same clade if they were part of the same community. I would not label this as “risk,” rather that the distribution of clades across communities was non-homogenous. Why not use odds/odds ratio instead of risk? It also would make sense to construct a 2x2 contingency table here, with same community (0/1) and same clade (0/1), and perform the Fisher’s exact test. However, they also performed a t-test, and it is unclear what that was testing. Clarification is needed.*

Answer: As the reviewer understood well, we aimed to determine if any two sequences in the same clade were also part of the same community. We used a Fisher exact test as suggested by the reviewer and calculated the Odds Ratio and 95% C.I. For almost all partitions, the OR estimated was higher than 1 and the confidence interval did not contain the value1, except for the Edge_Betwenness algorithm using the brockmann distance. We changed the table and the text to include the new estimations and remove the t-test (L278-285/600-604, Table 1).

- *L256-258. I am not entirely convinced that these “hotspots” are true hotspots based on simply having >1 clade identified. This should be backed up with statistics. The paper reports only 39 sequences across the network of hundreds of nodes. Thus, are nodes sufficiently sampled to determine the absence of other clades at poorly sampled nodes?*

Answer: We defined “hotspots” in a tentative manner, based on the available data. We agree with the reviewer that this identification could change if more nodes and more animals were sampled, however, in our case only animals presenting PPR symptoms were sampled opportunistically, thus reducing the possibility for large sample size. Although it is important to note that this data represents one of the most complete PPRV genetic datasets ever obtained from a single endemic region, and it is a major achievement in itself (as said in L326). To confirm our hypothesis regarding hotspots, further work with a larger dataset is needed. However, we still think that it is interesting to present the hypothesis of hotspots as a preliminary analysis in wait of further data, because such data may take a long time to come if not motivated by preliminary results. Nevertheless, the text in the results (L313-316) and discussions (L369-392) sections has been modified to stress the lack of evidence to support this hypothesis based on the data available.

- *L272-274. Here, the data shows that hotspot nodes have a tendency to form more within-community ties. However, is it important that they are within-cluster (homophily), or do the high homophily values simply reflect the higher overall degree of hotspot nodes. I suggest reporting both the strength of within-community and between-community ties to help tease apart degree, and within/between community ties.*

Answer: We would like to thank the reviewer for this suggestion. We checked the strength of the homophily based on the volume and the frequency of animals exchanged with other nodes in the same community. In most of the cases the tendency is confirmed and hotspot nodes apparently tend to exchange more and more often with nodes in the same community than monoclade nodes do. However these trends were not significant based on Kruskal -Wallis tests. Therefore, at this stage, we can only suggest that our data showed some trends but more data and analyses are needed.

Results of the Kruskal-Wallis tests have been added to the text (L313 and 618) with further notes of cautions regarding the preliminary nature of the analyses performed (see response to comment above).

- *L45: Revise to “significantly more likely”*

Answer: Done

- *L127: Change “completed” to “complemented”*

Answer: Done

- *L184: The phrase “at least 9 800 animals annually” falls awkwardly in the sentence. Revise.*

Answer: The sentence has been modified (L202-205).

- *L185. The authors write that many links occurred “once a year.” My understanding is that data was only collected for a single year, and “once a year” sounds as if the links are repetitive across years, which is not supported by the study design.*

Answer: The sentence has been modified (L202-205)

- *L194-195. This sentence is repetitive with the previous. I suggest deleting.*

Answer: The sentence has been deleted

- *L200. The authors first write that community size and structure differed based on weighting and algorithm. However, here they state the similarlity between community structure was high. Here, its not clear what is being compared.*

Answer: The similarity between communities is based on the Rand Index. For each possible pair of elements in the considered set, the Rand Index evaluates how similarly the two partitions treat them. Because of this, independently of size distribution, there could be a high degree of similarity between communities partition, meaning that pair of nodes that were members of the same community according to a partition scheme are likely to appear in the same community using other criteria.

The sentence has been rewritten (L218-2019) and an explanation of the Rand Index has been added in the Material and method section (L590-593).

- *L209. Revise to “others”*

Answer: Done

- *L210. Define brockman distance.*

Answer: The Brockmann distance was already defined in the methods (L515-518), but a short definition is now also given in the results section (L230).

- *L216. What is included in “other distance.” Spatial distances, or network-related distance?*

Answer: Both. The sentence has been changed accordingly (L229)

- *L219. The Mantel correlation between genetic distances and what?*

Answer: The mantel correlation between genetic distances and the spatial and network-related distances. We changed the sentence accordingly (L236).

- *L232. Revise to “the largest community detected by the Edge_Betweenness algorithm, was not spatially clustered regardless of weighting”*

Answer: Done

- *L243. The authors should use Spearman correlation for this.*

Answer: We would like to thank the reviewer for the suggestion. We used the Spearman correlation for both algorithms and all criteria. Excepted for the InfoMap case weighted by frequency, all the correlation coefficients were positive and significative at least at 10%. We changed the results section accordingly (L279-286).

- *L269. There is no Table S9. Additionally, the text implies that frequency is an incoming metric, but that is unclear in Table S4. In addition, hotspot nodes have higher indegree, but not incoming frequency?*

Answer: We corrected table S9 to Table S6 (former Table S4). A link exists between 2 locations if at least an animal is exchanged between the two. We can characterize the importance of the link using several properties of the movement: we can use the total volume of animals exchanged between the two locations during a year (Volume) ; we can use the number of times a link is active, that is how many times animals have been exchanged between the two ends of the link independently of the volume exchanged (frequency). Since we are dealing with oriented network, when we are estimating network properties we have to distinguish between incoming and outgoing properties. Incoming degree of a node accounts for the number of connections pointing to the node; incoming frequency accounts for the frequency of movements toward the node. Because of this, a node could be the destination of movements from a large number of nodes (high indegree) but these movements can seldom occur (low incoming frequency). We are mostly interested to incoming movements, since these could be the main way to introduce strains in a specific area. The Tables S6 and S7, and the text (520-521) have been modified to specify that frequency component concerns frequency of incoming movements only.

- *L269. Comparison of node metrics should be backed up with a statistical test (Kruskal-Wallis would be appropriate).*

Answer: A Kruskall-Wallis test has been done for each node metric, see responses to comments above.

- *Tables S4-S5. Check numbering. The text and the numbering may not be consistent? Or perhaps the text in the results needs greater clarification to understand how the results relate to the tables. For example, L275 says that Table S5 is a discriminant analysis… however it appears from the table captions that S5 is hierarchical clustering and S4 is a linear discriminant analysis.*

Answer: The numbering of the tables has been corrected.

- *L294-294. The authors report multiple co-circulating clades. While I agree with their previous description that these likely represent common transmission chains and a recent common ancestor, it would be helpful to know what the genetic distance was for these clades.*

Answer: Information on genetic distances have been added at L176-179. Pairwise genetic distance separating samples within clusters (mean distance: 0.30%) was significantly lower than sequence distance between samples from different clusters (mean distance: 0.52%; Wilcoxon test, W=10823, *p* < 0.0001).

- *L296. “Phylogenetic relationships between clades were unclear.” Is this because of low bootstrap support?*

Answer: Yes, nodes had only limited posterior probability and boostrap support, preventing us from doing any inference on the phylogenetic relationships between clades.

- *L428-429. Its unclear why clades were identified using both ML and a Bayesian method. I would have expected that final inference would be done with one or the other (with ML often a precurser to Bayesian analysis). The authors should elaborate more on this.*

Answer: All inference methods have their bias. We believe that using multiple methods improve confidence on node support in phylogenetic analyses. This methodology is quite common, see for example:

<https://doi.org/10.1371/journal.ppat.1002094>

<https://doi.org/10.1073/pnas.0901308106>

<https://doi.org/10.1016/j.ympev.2012.08.019>

Explanations have been added in the methods section (L478).

- *L448. Remove the word “complex.” The SNA approach appears to be a standard weighted network.*

Answer: We believe that the term “complex” is appropriate in this context. The term social network usually refer to individuals and their social relations. Complex network or complex network analysis instead refer to the mathematical/physics branch that has interest in analysing large networks. Complex network has connection on one side with social network and on the other to graph theory.

- *L472-473. It appears that viral sequences were assigned, in some cases, to the closest node for which there was data in the network. The median distance corresponded to 1-2 days of walking. How sure are the authors that animals that far away may not go to a different node? At the very least, this assumption should be mentioned in the discussion.*

Answer: Our mobility dataset does not contain enough information about small range and small size movements, corresponding to small livestock owners movements. However, based on information on the structure of the value chain in Senegal (see reference 35 in the text), the majority of small livestock owners go to the weekly market in the vicinity to sell/buy animals and in most of the cases this type of movements are done by walking. To have a better description of market’s catchment area, ad-hoc activities should be put in place to collect information about owners’ market choices and distance walked. We added few lines to explain this point in the methods section (L536-538) and in the discussion (L389-393).

*L505-506. “The assumption to test was if communities are monoclade or a hotspot.” Revise for clarity.*

Answer: The sentence has been rewritten as “We tested the hypothesis that two sequences were more likely to be in the same clade if they were in the same community…” (L601-602)
