# Supplementary material for: Combining viral genetic and animal mobility network data to unravel peste des petits ruminants transmission dynamics in West Africa
Source: PLoS Pathog. 2021 Mar 18;17(3):e1009397. doi: 10.1371/journal.ppat.1009397 (PMC8009415; doi:10.1371/journal.ppat.1009397)
Supplement: Attachment — Submitted filename: Response to reviewer-revision2-final.docx [file ppat.1009397.s023.docx]

- *On Line 199-201, you write “Both the indegree and outdegree distributions followed a power law indicating the presence of a few highly connected nodes (hubs) and many poorly connected nodes (Figure S3).” You do not report the power law coefficient and you do not seem to have mentioned the fitting procedure in the methods. Please add this information. Also, you have not tested statistically for power law behaviour of your degree distributions. The plot might also be interpreted as this behaviour not being necessarily obvious. Please do so or nuance your statement.*

The indegree and outdegree distribution behavior were assessed using a log-log fit of the distributions against the indegree and outdegree. The fit provided the values for the coefficients (with p-value<0.001) for the 2 distributions, as well as values for R^2^ (around 0.8). The coefficients are reported in the article together with the formulas for the distribution (lines 200-206):

“Both the indegree ($k_{in}$) and outdegree ($k_{out}$) distributions ($P\left( k_{in} \right),P\left( k_{out} \right)$, respectively) followed a power law

$$P\left( k_{in} \right)\sim k_{in}^{{-\alpha}_{in}},\alpha_{in}=1.188 \left( p value<0.001 \right), R^{2}=0.808;$$

$$P\left( k_{out} \right)\sim k_{out}^{{-\alpha}_{out}},\alpha_{out}=1.309 \left( p value<0.001 \right), R^{2}=0.842$$

indicating the presence of a few highly connected nodes (hubs) and many poorly connected nodes (Figure S3).”

In the “Material and Methods” section we added a sentence explaining the fitting procedure (lines 524-528):

“For all the nodes we estimated some centrality measures. More specifically, we estimated for each node the number of incoming links (indegree $k_{in}$ ) and outgoing connections (outdegree $k_{out}$) and the distributions of these measures all over the network. For both distributions, we performed a log-log fit to check whether the distribution was following power law.”

- *On line 234-6, you wrote “Only genetic distances between pairs of sequences within well-defined phylogenetic clades were used for these analyses, as they represented groups of viruses originating from a common chain of transmission events.” It would be useful to mention how many pairs are considered.*

118 pairs of sequences were used for the analysis. The information has been added at line 239.

- *Likewise, for the Mantel correlogram analysis, how many pairs are included under each class?*

The number of classes used was automatically determined using Sturge’s law, to obtain a smoother partition of data (i.e. not choppy or discontinuous and neither too large to mask variations). Instead of detailing the number of pairs in each class we summarized the distribution of nodes in the methods section (lines 579-581):

“The number of classes was determined using the Sturge’s law, producing 7 small classes (each containing less than 8 pairs) and 4 large ones (containing between 10 and 31 pairs). The first 2 classes contained half of the pairs.”

- *It is unclear how the hotspot analysis is influenced by the number of sequences available in each node. It would be helpful for the readers to summarise the distribution of sequences/site, especially the number of sites where multiple sequences are available, and whether being a hotspot vs monoclade site is associated with the number of sequences available per site.*

A new table has been added in the Supplementary Table (Table S6) containing for each node the number of sequences available and if it is categorized as a Hotspot or not. The table has been cross-referenced along the work. Concerning the second point, we run a Wilcoxon test checking if the number of sequences available in Hotspots was higher than in monoclade locations. The test was significative (p-value <0.05). This could suggest that increasing the number of strains could result in more nodes to be hotspot, and our definition could be related to the limited number of collected samples. Then, we included the number of sequences as other possible explaining factor. In the linear discriminant analysis the effect of the available strain is positive but smaller compared with network measure. However, these conclusions were not statistically significant (Kruskal -Wallis test, p-values > 0.05). To support this conclusion we also performed logistic regression using the same data. The results were coherent with the linear discriminant and not statistically significantWe explain this point in the material and methods section (lines 632-640):

“We used the terms hotspot to indicate nodes where PPRV sequences of different clades were found, while we used the term monoclade to indicate nodes where all the sequences available belonged to the same clade (Table S6). We used linear discriminant analysis, using the nodes’ network characteristics, to identify the factors that characterise hotspots. In a preliminary analysis we found that more sequences were collected in hotspot nodes compared to monoclade ones, and the limitedness of the sampling procedure could result in misclassification of nodes. To take this into account, we considered the number of sequences available as a possible factor that could characterise hotspot. Then, we included other possible factors, related to livestock trades and mobility, which could help identifying possible hotspot locations.”

And in the result section (Line 323-328):

“Discriminant analysis showed that the homophily was the variable with the highest probability of classifying a node as a hotspot (Table S8) while high centrality (eigenvector centrality) had a considerable negative effect. The small sampling size (22 nodes among which only four are hotspots, all with a small number of animals sampled Table S6) had a significant effect on identifying hotspots (Wilcoxon test p-value <0.05), suggesting that hotspots were more likely to be identified in nodes containing more sequences, but its effect, in most of the cases, was smaller (in magnitude) when compared to abovementioned measures (Table S8).”

- *On line 612, you wrote “odds ratio was then computed for the two groups using Fisher Exact test.” This is confusing as you do not need to conduct Fisher exact tests in order to compute Odds Ratio. To avoid confusion, please specify explicitly the actual equations of the odds ratios you computed.*

Odds ratio were estimated using the usual formula while we used Fisher exact test to estimate p-value and Confidence Interval of the odds ratio. The methods section has been modified to clarify this point (line 627-629):

“The odds ratio was computed for the two groups and the Fisher Exact test used to check whether the OR was different from 1 and to estimate its p-value and 95% Confidence Interval.”

- *On Table S6, hotpost and monoclade are defined based on the number of clades to which the sequences sampled in those sites belong. The purpose of the hierarchical clustering analysis mentioned in the title of Table S6 is therefore unclear.*

The presence of one or more clades in a location has been used to define Monoclade or hotspots. However, we tried to identify the factors that could explain why strains of different clades are concentrated in a location, i.e. profiling those nodes. Further explanations have been added to the notes of Table S7 :

“Homophily, i.e. the tendency to create connections with members of the same community, is estimated for each community partition, and for different types of strength of connection. All these characteristics are considered as possible “predictors” to identify hotspot locations, i.e. factors that could justify the presence of strains of more than a single clade.”

- *On line257, specify the actual range of distances for both subsets.*

Done (lines 261-262)

- *Table S4 caption: “distance distances” remove “distance”*

Done

- *On line 215, you refer to Figure S4, should it be Figure S5?*

The figure number has been corrected (line 218).

- *On line 267, “closely related PPRV strains in the same clade” replace “in” by “of”*

Done (line 271)

- *The Figure numbers are missing in several places (Line 275, 281, 283, 298). Please check your manuscript carefully*

The figure numbers have been checked and corrected. The error was related to the use of the automatic cross reference tools in Word.

- *Line 332-334 "This is one of the most complete PPRV genetic datasets ever obtained from a single endemic region" - I appreciate that PPRV sequences are scant, and the authors have taken commendable efforts in generating the sequence data, but given that the size of the dataset is small and the likely sample bias, this statement is a bit misleading or at the very least exaggerated. I think the authors should emphasize the fact that by using complete N and H sequences, with a combined length ~3500, they had sufficient statistical power to detect distinct clades within lineage II. I believe this is a particular strength of the study, as it is unlikely that the authors would have been able to obtain the same insights if they used short gene sequences.*

The statement has been removed and replace by (line 342-344):

“The length of sequences generated (combined length of N and H genes = 3402bp) provided enough statistical power to detect distinct clades within lineage II circulating concurrently in the region.”

- *Figure 1 - the inset figure is not easy to read. In particular, I could not discern the black and turquoise diamonds. Based on the high-res figure, should the turquoise diamonds be grey?*

The figure (Figure 3) has been modified to improve the clarity of the inset.

- *Figure 7 isn't mentioned in the main text (I suspect this may be related to my first point). I also have issues with the presentation of the figure. I found the text size in this figure too small and as a result challenging to read. Furthermore, compared to the country maps presented in Figures 2, 5, and 6, the map in Figure 7 seems elongated along the latitude axis.
  To simplify this figure, I would suggest colouring in each region depending on whether there are multiple or single lineages detected, instead of using small points. If you want to highlight the hotspots in more detail, you could present a zoomed-in version for these locations.*

We thank the reviewer for the suggestions. The missing figure references were due to a problem with the automatic cross-reference in Word and the upload process. Now it has been fixed.

We modified the figure to scale correctly the map so to have the same proportion between longitude and latitude axes. We increased the font size to improve clarity. We followed the suggestion of coloring the Administrative Units were hotspots nodes were located and labelled them. We also included the names of the region they belong to. We eliminate the locations of collected sequences

**“Figure 7. Geographical locations of hotspots and monoclade**. Hotspots and monoclade are geolocalised and the wards (Administrative Unit of level 3) to which they belong are colored according to the type of node (hotspot or monoclade). Nodes are scaled based on their incoming volume of animals. Labels in uppercase letters indicate node names. Labels in lowercase letters correspond to the names of regions in Senegal.”
