# Supplementary material for: Combining viral genetic and animal mobility network data to unravel peste des petits ruminants transmission dynamics in West Africa
Source: PLoS Pathog. 2021 Mar 18;17(3):e1009397. doi: 10.1371/journal.ppat.1009397 (PMC8009415; doi:10.1371/journal.ppat.1009397)
Supplement: Attachment — Submitted filename: Response to reviewer-revision3.docx [file ppat.1009397.s024.docx]

- *Figure 7: The latitude axis is still elongated.*

The figure has been modified.

- *Hierarchical clustering in Table S7: this is still unclear, as it seems that Table S7 only provides average values of statistics for two pre-defined sets of nodes, and no hierarchical clustering analysis has actually been done. If you did a hierarchical clustering analysis, please explain it in the methods.*

The title of Table S7 has been changed to

“Table S7. Average values of network characteristics in hotspot and monoclade nodes”

And this sentence has been added to the footnote of Table S7:

“Values in parentheses represent standard deviation”

Moreover in the text at line 392 we changed “hierarchical clustering” to “linear discriminant”

- *Table S7: in addition to average values, please provide a range or standard deviations.*

Standard deviation values for all coefficients have been added in parentheses

- *There seems to be an issue with the formatting of Tables S7-8, please edit these.*

The tables have been re-formatted
